# Southward Expansion of *Ixodes scapularis* (Blacklegged) Tick Pathogens — Western North Carolina, November 2024–August 2025

**DOI:** 10.15585/mmwr.mm7531a1

**Published:** 2026-08-13

**Authors:** Sean Sweeney, Ayla Bullock, Michael Reiskind, Angela Newnam, Jonathan Kanipe, Melissa S. Nolan, Ross M. Boyce, North Carolina Biltmore Forest Study Team

**Affiliations:** ^1^University of South Carolina, Columbia, South Carolina; ^2^University of North Carolina at Chapel Hill, Chapel Hill, North Carolina; ^3^North Carolina State University, Raleigh, North Carolina; ^4^Biltmore Forest LEADS Task Force, Biltmore Forest, North Carolina.

**Summary**

**What is already known about this topic?**

Lyme disease, the most frequently reported vectorborne disease in the United States, is emerging beyond the regions of the Northeast and upper Midwest where it has historically been considered endemic, with increasing transmission in southern Appalachia.

**What is added by this report?**

Community-based tick surveillance conducted at residential properties in the town of Biltmore Forest, North Carolina, identified blacklegged ticks (*Ixodes scapularis*) as the predominant tick species. Approximately 40% of adult *Ix. scapularis* tested positive for *Borrelia burgdorferi* sensu stricto, the bacterium that causes Lyme disease. *Borrelia miyamotoi* and the human-active strain of *Anaplasma phagocytophilum* were also detected in collected *Ix. scapularis* specimens.

**What are the implications for public health practice?**

Western North Carolina is an area of emerging risk for Lyme disease and other tickborne infections. Enhanced clinical awareness, public education about tick bite prevention, and expanded tick and pathogen surveillance are needed to improve early diagnosis and reduce transmission of tickborne infections in southern Appalachia, a region where Lyme disease is emerging but not yet considered endemic.

## Abstract

Southern Appalachia is a region where Lyme disease is emerging but not yet considered endemic. In response to local concerns about increased numbers of tickborne disease cases, researchers collected ticks monthly at 22 residential properties in the town of Biltmore Forest, North Carolina. During November 2024–August 2025, a total of 373 ticks were collected, 287 (76.9%) of which were blacklegged ticks (*Ixodes scapularis*). Among these, 19 of 48 adult *Ix. scapularis* (39.6%) tested positive for *Borrelia burgdorferi* sensu stricto, the bacterium that causes Lyme disease. *Borrelia miyamotoi* and *Anaplasma phagocytophilum* were also identified in collected *Ix. scapularis* ticks, suggesting an expansion of the geographic range of these ticks and pathogens. These findings illustrate the evolving epidemiology of tickborne infection risk and the need for enhanced tick surveillance in southern Appalachia.

## Introduction

Historically, Lyme disease, caused by infection with the bacterium *Borrelia burgdorferi*, has been geographically associated with the Northeast and upper Midwest regions of the United States. However, in recent years, an increasing number of Lyme disease cases have been reported in the southeastern United States, particularly along the Appalachian Mountains range (*1*). In 2024, researchers partnered with a community task force in Biltmore Forest, a heavily wooded town located in the Blue Ridge Mountain region of North Carolina, after community members raised concerns about an increase in numbers of tickborne diseases. Biltmore Forest spans approximately 1,600 acres, located between the Biltmore Estate and Asheville, North Carolina. This report describes the epidemiologic investigation into ticks and tickborne disease in an area where Lyme disease is rapidly emerging but not yet considered endemic.

## Methods

### Community Survey and Follow-Up

In early 2024, the community task force developed and mailed surveys to the approximately 600 residential households within the town of Biltmore Forest; 75 (12.5%) responses were returned. This survey documented any history of Lyme disease in the responding household, as well as frequency of tick encounters, use of tick bite prevention measures, and general knowledge about tickborne diseases. The research team partnered with the University of North Carolina at Chapel Hill, North Carolina State University, and the University of South Carolina, obtained institutional review board approval, and then received the completed surveys for analysis. Study investigators contacted households reporting persons with tickborne disease cases by telephone to obtain additional information related to their diagnosis, including any available medical records. During these calls, several respondents voluntarily referred investigators to other persons known to have received a diagnosis of a tickborne disease but who were not included in the initial community survey. These persons were also contacted and provided data subsequently included in the analyses. This activity was reviewed by CDC, deemed not research, and conducted consistent with applicable federal law and CDC policy.^†^

### Tick Specimen Collection

Permission was obtained from 22 survey respondents to establish tick surveillance at their residential properties. Ticks were collected using distance dragging/flagging with 1.0 m × 1.5 m (3.3 ft x 4.9 ft) duck/flannel cloth over a drag area of 300–500 m^2^ (3,229–5,381 sq ft) per property. Monthly drags were conducted during November 2024–August 2025. The collection scheduled for May 2025 was canceled because of adverse weather. Willing residents, irrespective of their participation in the community survey or monthly property drags, were also provided kits with instructions for collecting any ticks they discovered on themselves, their pets, or their properties. Collected ticks were sent to the study team or given to investigators during their visits to the town. All specimens were stored in ethanol and transported to the University of South Carolina for DNA extraction. Ticks were identified by species and life stage using a morphological dichotomous key, and DNA was extracted using previously described methods (*2*). Each adult and nymphal (immature) ticks were processed and tested individually. Larval ticks underwent DNA extraction and testing in aggregate, a process known as pooling. Pooling involves placing multiple individual larvae in a single tube, extracting their DNA in aggregate, and testing the extracted DNA as a single sample. Larvae were pooled by property location, date, time, and species. Larval pool sizes ranged from six to 149 larvae per pool.

### Pathogen Testing

Extracted DNA samples from *Ixodes* spp. ticks were shipped to CDC’s Division of Vector-Borne Diseases in Fort Collins, Colorado, where they underwent pathogen testing via multiplex polymerase chain reaction amplicon sequencing as part of the national tick and tickborne disease pathogen surveillance program (*3*). Pathogens eligible for testing included *Babesia microti*, *Ehrlichia muris eauclairensis*, *Anaplasma phagocytophilum* human-active strain, *A. phagocytophilum* nonhuman-active strain, *Borrelia mayonii*, *Borrelia miyamotoi*, and *B. burgdorferi *sensu stricto.

## Results

### Self-Reported Human Disease

In addition to the 75 household survey responses obtained in 2024, seven additional self-reports from seven unique households were captured through referral by other community members. In total, 19 persons from 17 different households reported at least one tickborne disease diagnosis while living in Biltmore Forest. The most frequently reported illness was Lyme disease (17 cases); two cases of babesiosis and one case of ehrlichiosis. Two persons reported coinfection with two distinct pathogens, and one person reported having received a diagnosis of a tickborne disease but could not recall the specific disease. Four of the 19 persons (21.1%) who reported illness provided medical records; among these persons, one had laboratory evidence of *B. burgdorferi* infection, two had documentation of erythema migrans after a tick exposure, and one had no documented evidence of infection. Diagnosis was based on self-report for the remaining 15 persons.

### Tick Collection, Identification, and Pathogen Testing

A total of 373 ticks were collected from 25 properties during the study. Of these, 337 (90%) were collected through monthly drags from 22 properties. An additional 36 ticks were collected by residents. Four of these ticks came from three properties that did not participate in the monthly drags (Supplementary Figure). Most ticks (287; 76.9%) were identified as *Ixodes scapularis* (Table 1). Five other species were also identified: *Dermacentor variabilis* (34 ticks), *Amblyomma americanum* (five), *Ixodes dentatus* (five), *Haemaphysalis leporispalustris* (four), and *Haemaphysalis longicornis* (one). Overall, 73.7% of ticks were larvae, 23.6% were adults, and 2.7% were nymphs. Damaged morphologies inhibited morphologic identification of 37 ticks. Most ticks (315; 84.5%) were collected during July–November.

**TABLE 1:** Tick species collected from 25 residential properties — Biltmore Forest, North Carolina, November 2024–August 2025

Tick species	No. (%)			
	Larvae n = 275	Nymphs n = 10	Adults n = 88	Total N = 373
*Ixodes scapularis*	230 (83.6)	7 (70.0)	50 (56.8)	**287 (76.9)**
*Ixodes dentatus*	3 (1.1)	1 (10.0)	1 (1.1)	**5 (1.3)**
*Haemaphysalis leporispalustris*	3 (1.1)	1 (10.0)	0 (—)	**4 (1.1)**
*Haemaphysalis longicornis*	0 (—)	0 (—)	1 (1.1)	**1 (0.3)**
*Dermacentor variabilis*	0 (—)	0 (—)	34 (38.6)	**34 (9.1)**
*Amblyomma americanum*	3 (1.1)	0 (—)	2 (2.3)	**5 (1.3)**
Unknown	36 (13.1)	1 (10.0)	0 (—)	**37 (9.9)**

Six ticks (1.6%) were excluded from testing because of desiccation or improper transport conditions. Among 292 collected *Ixodes* spp., a total of 286 were suitable for testing including 49 adults, eight nymphs and 229 larval ticks aggregated into nine larval pools (Table 2). Nearly two in five (39.6%) *Ix. scapularis* adults were positive for *B. burgdorferi *ss. In addition, three of seven (42.9%) *Ix. scapularis* nymphs were positive for *B. burgdorferi *ss. Positive specimens were widely distributed across the town. No larval pools were positive for *B. burgdorferi *ss. Another *Borrelia* species, the relapsing fever spirochete *B. miyamotoi,* which is known to infect the eggs of adult female ticks before they are laid, was detected from two *Ix. scapularis* adults and two aggregated larval pools. In addition, *Ix. scapularis* adults were found to carry both the human-active strain (four adults) and nonhuman-active strain (one adult) of *A. phagocytophilum*. The nonhuman-active strain (also known as *A. phagocytophilum* variant 1) is found in *Ix. scapularis* but does not cause disease in humans (*4*). The density of ticks infected with at least one tickborne pathogen follows a similar geographic distribution to the density of self-reported tickborne disease cases (Figure).

**TABLE 2:** Pathogen prevalences in *Ixodes* spp. tick pools — Biltmore Forest, North Carolina, November 2024–August 2025*^,†^

Pathogen species	Larvae^§^		Nymphs**		Adults^††^	
	No. (% positive)	95% CI^¶^	No. (% positive)	95% CI^¶^	No. (% positive)	95% CI^¶^
*Anaplasma phagocytophilum* (human-active strain)	0 (—)	NA	0 (—)	NA	4 (8.3)	2.7–20.8
*A. phagocytophilum* (nonhuman-active strain)	0 (—)	NA	0 (—)	NA	1 (2.1)	0.1–12.5
*Borrelia burgdorferi*	0 (—)	NA	3 (42.9)	11.8–79.7	19 (39.6)	26.0–54.6
*Borrelia miyamotoi*	2 (25.0)	4.4–64.4	0 (—)	NA	2 (4.2)	0.7–15.5

**Abbreviation**: NA = not applicable.

* No larval pools or individual adults or nymphs of any tick species tested positive for the following pathogens: *Ehrlichia muris eauclairensis*,* Babesia microti*, or* Borrelia mayonii.*

^†^ No *Ixodes dentatus* pools tested positive for any pathogen.

^§^ Nine pools; pool size range = six–149 ticks.

^¶^ 95% CIs were calculated using the Wilson score method.

** Eight pools; pool size = one tick.

^††^ Forty-nine pools; pool size = one tick.

**FIGURE:**
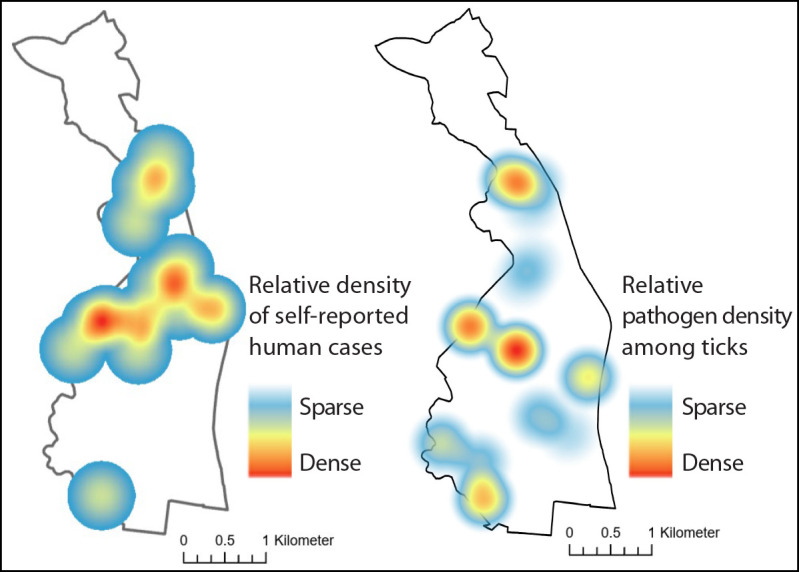
Relative density of self-reported human cases* of tickborne disease and relative pathogen^†^ density among collected ticks^§^ — Biltmore Forest, North Carolina, November 2024–August 2025 * Locations reflect approximate location of residence of persons who reported having had one or more tickborne disease diagnoses at any time while living in Biltmore Forest. ^†^ *Borrelia miyamotoi*, *Borrelia burgdorferi *sensu stricto, and *Anaplasma phagocytophilum*. ^§^ Ticks were collected during November 2024–August 2025.

## Discussion

The prevalence of *B. burgdorferi *ss, together with the presence of *B. miyamotoi* and *A. phagocytophilum* identified in adult and nymphal blacklegged ticks, highlights the increasing tickborne disease risk in southwestern North Carolina. The prevalence of *B. burgdorferi *ss identified in adult blacklegged ticks collected in Biltmore Forest is similar to the prevalence in the mid-Atlantic, upper Midwest, and Northeast, where it is endemic (*5*). Previously reported *B. burgdorferi* infection prevalences among *Ix. scapularis* collected from public lands in Buncombe County, where Biltmore Forest is located, range from 13% to 17% in nymphs to 25% in adults; because ticks were collected exclusively from residential areas in this study, the high infection prevalence identified in this report suggests higher potential for human disease than that identified in previous studies (*6*). However, surveys in neighboring South Carolina counties found low prevalence of infection with *B. burgdorferi* among *Ix. scapularis*, suggesting that the Biltmore Forest study area represents the leading edge of southward expansion of northern populations of *Ix. scapularis* (*7*). In addition, capture of host-seeking *Ix. scapularis* nymphs in this study indicates the presence of northern clade ticks in this southeast state (*8*).

These findings might represent the farthest south that human-associated variant *A. phagocytophilum* and *B. miyamotoi* have been identified in *Ix. scapularis* populations (*9*) and highlight the potential for pathogen transmission in a new geographic area. Given that both *A. phagocytophilum* and *B. miyamotoi* can cause severe clinical disease, efforts to raise awareness in this region are needed, both to help guide clinicians’ diagnostic decisions and to encourage self-protective measures such as use of insect repellent and protective clothing (e.g., long pants and tall socks).

### Limitations

The findings in this report are subject to at least five limitations. First, specimen collection began shortly after Hurricane Helene, which resulted in ecological destruction in western North Carolina. Without comparable data from before the storm, it is unclear how this hurricane might have affected regional tick populations. Second, because tick collection occurred during 1–3 days each month, collections were subject to variable environmental and weather conditions that might have affected the presence or absence of host-seeking ticks on the day of collection. This timeframe limits the ability to characterize the temporal presence of ticks in the study area. Third, a relatively small area of the town was dragged for ticks, limiting the ability to determine tick densities. Fourth, because the survey achieved a relatively low response rate of 12.5%, the results carry a heightened risk for selection bias. Thus, the results of this survey reflect a small subset of the population of Biltmore Forest and might not be representative of the entire community. Finally, because the majority of human tickborne disease cases were identified through self-report, these data are subject to potential misclassification.

### Implications for Public Health Practice

Given the high pathogen prevalence of *B. burgdorferi* ss in *Ix. scapularis*, as well as the identification of *B. miyamotoi* and *A. phagocytophilum,* increased clinical suspicion for human disease attributable to these pathogens among those who live in or visit this region is warranted. A public health alert for health care providers who have a high likelihood of encountering patients with exposure to tickborne disease (e.g. primary care, urgent care, and infectious disease practitioners), many of whom do not have experience with the diagnosis and management of Lyme and other tickborne diseases (*10*), could help disseminate this new information. Expansion of *Ix. scapularis* surveillance and associated pathogen testing in the broader southeastern United States is also warranted. Education for the public to prevent tick bites includes wearing appropriate protective clothing, using Environmental Protection Agency–registered insect repellents, and checking the body for ticks after spending time outdoors. Guidance for preventing tickborne disease can be found online. 

## Footnotes

† 45 C.F.R. part 46; 21 C.F.R. part 56; 42 U.S.C. Sect. 241(d), 5 U.S.C. Sect. 552a, 44 U.S.C. Sect. 3501 et seq.
